# Knowledge, attitudes and practices of female genital mutilation/cutting among health care professionals in The Gambia: a multiethnic study

**DOI:** 10.1186/1471-2458-13-851

**Published:** 2013-09-16

**Authors:** Adriana Kaplan, Suiberto Hechavarría, Mariola Bernal, Isabelle Bonhoure

**Affiliations:** 1 Chair of Social Knowledge Transfer/Parc de Recerca UAB - Santander, Department of Social and Cultural Anthropology, Universitat Autònoma de Barcelona, Barcelona, Spain; 2 Interdisciplinary Group for the Prevention and Study of Harmful Traditional Practices (IGPS/HTP), Department of Social and Cultural Anthropology, Universitat Autònoma de Barcelona, Barcelona, Spain; 3 NGO Wassu Gambia Kafo, Fajara F Section, Banjul, The Gambia; 4 Cuban Medical Mission in The Gambia, Banjul, The Gambia; 5 Community-based Medical Programme, Ministry of Health and Social Welfare, Banjul, The Gambia; 6 Facultad de Ciencias Médicas Manuel Fajardo, Universidad Médica de La Habana, Havana, Cuba

**Keywords:** Female genital mutilation/cutting, The Gambia, Health care professionals, Knowledge, Attitudes and practices (KAP), Sexual and reproductive health, Africa

## Abstract

**Background:**

Female genital mutilation/cutting (FGM/C) is a harmful traditional practice with severe consequences for the health and well-being of girls and women. Health care professionals (HCPs) are therefore expected to be aware of how to identify and manage these consequences in order to ensure that those affected by the practice receive quality health care. Moreover, their integration and legitimacy within the communities allow them to play a key role in the prevention of the practice. Nevertheless, the perception of HCPs on FGM/C has been barely explored in African contexts. This study seeks to contribute to this field of knowledge by examining the knowledge, attitudes, and practices regarding FGM/C among HCPs working in rural settings in The Gambia.

**Methods:**

A cross-sectional descriptive study was designed through a quantitative methodology, following a multiethnic approach. A pre-tested questionnaire with open and closed-ended questions was created. Forty medical students from the Community-based Medical Programme were trained to administer the questionnaire, face to face, at village health facilities in rural areas of The Gambia. A final sample of 468 HCPs included all nurse cadres and midwives.

**Results:**

A significant proportion of Gambian HCPs working in rural areas embraced the continuation of FGM/C (42.5%), intended to subject their own daughters to it (47.2%), and reported having already performed it during their medical practice (7.6%). However, their knowledge, attitudes, and practices were shaped by sex and ethnic identity. Women showed less approval for continuation of FGM/C and higher endorsement of the proposed strategies to prevent it than men. However, it was among ethnic groups that differences were more substantial. HCPs belonging to traditionally practicing groups were more favourable to the perpetuation and medicalisation of FGM/C, suggesting that ethnicity prevails over professional identity.

**Conclusions:**

These findings demonstrate an urgent need to build HCP’s capacities for FGM/C-related complications, through strategies adapted to their specific characteristics in terms of sex and ethnicity. A culturally and gender sensitive training programme might contribute to social change, promoting the abandonment of FGM/C, avoiding medicalisation, and ensuring accurate management of its health consequences.

## Background

According to the World Health Organisation (WHO) [1], female genital mutilation/cutting (FGM/C) includes all procedures involving partial or total removal of the external female genitalia, or injury to the female genital organs, for non-therapeutic reasons. FGM/C is recognised internationally as a violation of the human rights of girls and women, constituting an extreme form of gender discrimination with documented health consequences. The WHO estimates that 140 million women and girls in the world have been victims of some form of FGM/C, and that each year, 3 million girls are subjected to, or at risk of being subjected to, this harmful traditional practice.

FGM/C has been classified by the WHO into four types. Types I (clitoridectomy), II (excision), and III (infibulation) are ordered according to a growing level of severity, while type IV comprises all other harmful procedures performed on the female genitalia for non-medical purposes (e.g. pricking, piercing, incising, scraping, and cauterisation). All types of FGM/C have consequences that undermine the health and well-being of newborns, girls, and women, exposing a situation that deserves attention in the world’s sexual and reproductive public health agenda. In the short-term, FGM/C might lead to shock, haemorrhage, infections, and psychological consequences, while in the long-term, it can cause chronic pain, infections, keloids, fibrosis, primary infertility, an increase in delivery complications, and psychological sequela/trauma [2-7]. In The Gambia, the impact of FGM/C was assessed through a recent clinical study conducted by the authors of the present study, where we found that one out of three girls and women suffered injuries as a consequence of this practice [8].

FGM/C is mainly performed in 28 countries in sub-Saharan Africa and in parts of the Middle East and Asia. However, FGM/C is also found in Europe, Australia, and the USA, to where migrants brought their culture. The origin of FGM/C is unclear, but this practice has been surrounded with a complex symbolic meaning. In many societies, FGM/C has become the physical proof that confirms that a girl has been initiated through a rite of passage to adulthood, confirming her femininity and ensuring that she has received all the necessary teachings to be worthy to belong in the community. Those who defend FGM/C perpetuation argue that it is critical to preserve ethnic and gender identity, protect femininity, ensure purity and virginity, guarantee the “family’s honour”, assure marriageability, and maintain cleanliness and health [9,10]. FGM/C is considered as a critical component of the process of socialisation and essential in the distinction between sexes as necessary opposites in the community, and it is linked with the two fundamental values that shape African life: sense of community and sex complementarity [11].

Sex complementarity does not necessarily mean gender equity. The oppression of women in patriarchal African societies has widely been considered as a major reason for the emergence and continuation of the harmful practice of FGM/C. In The Gambia, an estimated 76.3% of girls and women have been subjected to FGM/C, which is neither prohibited nor punished by law. With Islam being the predominant religion (90%) in The Gambia, women are submitted to customary and Islamic laws (Sharia). Their subordinate position in society is patent in their discrimination on issues, such as divorce or inheritance, inferiority in some legal aspects (e.g. the testimonies of two women are required to equalise the testimony of one man), and polygamy [12]. Moreover, they are subjected to high levels of domestic violence. A survey carried out in 2010 found that 74.5% of Gambian girls and women (15–49 years) believed that husbands were justified to beat their wives or partners for many reasons, including refusing to have sex, going out without telling them, or burning the food [13]. This concerning figure reveals how deeply girls and women are embedded in this patriarchal conception of the world.

FGM/C is performed on Gambian girls from 7 days after birth up to pre-adolescence, before the first menstruation and marriage [14-17]. Occasionally, adult women also subject themselves to this practice, as a consequence of peer pressure and intermarriage. Prevalence rates of FGM/C are significantly different between ethnic groups. The Gambia has an ethnically rich population, with different groups sharing cultural patterns due to historical relations, the small size of the country, generations of intermarriages, and the unifying force of Islam. Nevertheless, ethnic communities have also kept their own traditions, language, and background. For some of these communities, such as Mandinka, Sarahule, Djola, and Fula, this ritual has long been rooted in their cultural tradition. For others, such as Wolof and Serer, FGM/C is not a traditional practice as it can be confirmed when analysing these communities in Senegal, which do not perform it [16]. Consequently, discrepancies in prevalence rates are evident between Sarahule (97.8%), Mandinka (96.7%), Djola (87%), Serer (43%), and Wolof (12.4%) ethnic groups. Remarkable differences are also found between the prevalence rate of FGM/C in urban and rural areas. In the capital, Banjul, the prevalence of FGM/C is 56.3%, whereas in rural areas, such as Mansakonko (Lower River Region) or Basse (Upper River Region), the prevalence is almost universal (90.6% and 99.0%) [13].

The limited access to quality health care services, which is reflected by Gambia’s high rates of child^a^ and maternal^b^ mortality [18-20], affects the well-being of those experiencing FGM/C-related complications. This situation is particularly problematic in rural areas, where health care services are scarce, distances are long and often difficult to travel, communications are poor and, as mentioned above, the prevalence of FGM/C is higher than urban areas.

In these circumstances, health care professionals (HCPs) play an important role in FGM/C. Therefore, it is crucial to reach an accurate understanding of the knowledge, attitudes and practices (KAP) of HCPs towards the practice of FGM/C to envision effective strategies for their involvement. This has mostly been explored among western HCPs working in hospitals and primary health care centres located in receptor countries of African migrants, such as Spain, the UK, Switzerland, Sweden, Belgium, Australia, and the USA [21-28], indicating knowledge gaps concerning the health and legal issues of FGM/C.

Only a few studies have assessed this issue in an African context, and no studies have been conducted in The Gambia. In Nigeria, a study carried out by Onuh et al. [29] showed that medical knowledge on FGM/C was limited in nurses and their tendency was to support its continuation. Similar conclusions were obtained by Ragheb et al. [30] with nurses in Alexandria. In Egypt, Mostafa et al. [31] found that medical students were poorly informed about FGM/C-related complications, and that 52% of them believed that the practice should be maintained. Ali’s study [32], conducted among midwives in Eastern Sudan, showed that they did not consider the practice of FGM/C to be harmful and insisted on continuing it for cultural reasons.

These findings illustrate the importance of further exploring the KAP of HCPs in other countries where FGM/C is practiced to develop accurate and culturally sensitive methodologies for designing efficient training programmes. These programmes would be helpful for preventing this harmful traditional practice and for managing its consequences.

This study aimed to examine the KAP of FGM/C of Gambian HCPs working in rural settings. Our findings will also establish a baseline that can be used to compare and assess changes over time. We analysed differences among ethnic groups and sexes because they might be involved in KAP. Understanding these issues will enable a specific training programme to be designed, which could lead to an effective change.

## Methods

### Design of the study

To achieve the proposed objective, a cross-sectional descriptive study was designed to examine the KAP of Gambian HCPs regarding FGM/C. A multiethnic approach was chosen because this practice is closely linked to ethnic identity. The survey was conducted in 2009. Following a pilot study carried out in Banjul and the West Coast Region, implementation of the survey took place in rural settings throughout four regions: North Bank Region, Lower River Region, Central River Region, and Upper River Region.

The questionnaires were administered, face to face, by 40 medical students of the Community-based Medical Programme, under the Cuban Medical Mission in The Gambia. This research was conceived as part of their practicum in the Biostatistics course. The medical students attended 1 week of training on FGM/C and social research skills (including data collection, entry and analysis, as well as proposal development) by a team consisting of a medical anthropologist, a biostatistician, and a medical doctor. The organisation and supervision of the fieldwork were performed by members of the Regional Health Teams, under the authority of the Ministry of Health and Social Welfare. Their committed collaboration was fundamental to the implementation of this research.

The involvement of native interviewers was important for this study. This contributed to reduce the distance between interviewers and interviewees because they were both living in rural areas and shared the same culture. The interviews were conducted in English and/or local languages, as agreed between the interviewer and the interviewee. The involvement of native interviewers promoted the empowerment and ownership of knowledge of the native population, which was one of the core values of this research.

The interviewers were men and women, belonging to all ethnic groups. There was no matching between the sexes and the ethnic groups of interviewers/interviewees.

### Research population

The overall sample consisted of 468 HCPs, including nurses, community nurses, public health nurses, nurse attendants, and midwives. All public health system facilities located in the four rural regions were covered, which included hospitals, dispensaries, and major and minor health centres.

### Questionnaire and variables studied

The KAP questionnaire was designed by a medical anthropologist researcher with a thorough and extensive ethnographic background in The Gambia [16,33]. This questionnaire was designed following the implementation of barrier analysis using focus group discussions among Gambian men and women of all ethnic groups. Questionnaires were in English, the official language in the country. Four open and 25 close-ended questions were used to collect socio-demographic data (name of institution, occupation, age, sex, ethnic group, and date of interview) and on information regarding the KAP of HCP. The specifics of KAP were as follows. *Knowledge* on FGM/C health consequences (including exposure), reasons given for performing this practice, and its mandatory character in relation to religion, were examined. *Attitudes* towards the continuation of FGM/C, possible strategies for preventing it (including the role that can be played by HCPs and Islamic leaders), its medicalisation, the discrimination of girls who do not undergo FGM/C, and the involvement of men in the debate were examined. *Practices* included assessing if FGM/C is practiced in the HCP’s families/households, whether they would subject their own daughters to the practice, and whether they had ever performed FGM/C on girls.

The pilot study allowed testing of the consistency of the KAP questionnaire. The questionnaire was validated after being administered to 97 HCPs by medical students from the University of The Gambia.

### Ethical aspects

The study was approved by The Gambia Government/Medical Research Council Laboratories Joint Ethics Committee (Ref: R08002), which is the ethical committee in The Gambia. All respondents signed an informed consent, which was kept under the custody of Wassu Gambia Kafo, a non-governmental organisation that supported the study. Rigorous confidentiality over participants’ identity was maintained.

### Statistical analyses

Once collected, the data were computerised via Epidata and analysed in SPSS Version 19. Univariate and bivariate analyses with chi square tests were conducted to detect differences in KAP among HCPS of various ethnic origins and both sexes. Intra-sex and inter-sex relationships were tested. Statistical significance was considered at p < 0.05.

## Results

Table 1 shows the profile of the respondents. The final sample was composed of 468 HCPs (41.6% women and 58.4% men), with an average age of 28.8 years. The sample is ethnically representative of the total Gambian population, according to the most recently published official data [34].

**Table 1:** Profile of Gambian health care professionals (overall sample)

	**n**	**%**
**Total HCP respondents**	468	100
**Sex**		
Women	190	41.6
Men	267	58.4
Total	457	100
**Ethnicity**		
Mandinka	194	47.0
Djola	41	9.9
Wolof	60	14.5
Fula	81	19.6
Serer	22	5.3
Other	15	3.6
Total	413	100
	**n**	**Mean age**
**Age**	445	28.8

We found that a considerable proportion of HCPs (40.9%) observed girls and women with health complications resulting from the practice. Just under half of the respondents (42.5%) embraced its continuation and 7.6% reported to have performed it on girls. Detailed results in terms of KAP are presented in the following three subsections.

### Knowledge

The assessment of HCPs’ knowledge on FGM/C was performed by exploring the reasons given for the practice to be performed, as well as through acknowledging HCPs’ awareness of its health consequences. The results are shown in Table 2.

**Table 2:** Knowledge of FGM/C among Gambian health care professionals

	**Total HCP (%)**	**HCP by ethnic group (%)**					**HCP by sex (%)**	
		**Mandinka**	**Djola**	**Wolof**	**Fula**	**Serer**	**Men**	**Women**
**Reported answers of Gambian HCP about reasons/justifications given by those in support of FGM/C**								
It is a mandatory religious practice	53.8	54.9	43.8	42.6	58.7	60.0	56.3	48.8
It is a deeply rooted cultural practice	48.2	43	43.8	55.3	49.2	80.0	43.7	55.1
It reduces sexual feelings	42.1	41.5	56.3	36.2	47.6	40.0	43.7	38.6
It is a rite of passage for girls into womanhood	34.4	37.8	34.4	36.2	25.4	40.0	39.1	26.8
It is a good practice	23.3	28.7	15.6	19.1	25.4	6.7	26.1	18.9
It helps to maintain their virginity for their husband	13.5	14.1	12.5	8.5	14.3	6.7	12.6	15.7
It reduces the rate of prostitution	11.1	12.7	12.5	8.5	6.3	0.0	14.6	5.5
It does not violate human rights	0.6	0.0	3.1	2.1	0.0	0.0	0.5	0.8
**Reported answers of Gambian HCP about consequences on health of FGM/C**								
Transmission of infectious diseases	59.1	52.8	67.7	60.4	55.7	73.3	57.6	42.4
Bleeding	53.4	51.2	67.7	45.8	45.9	80.0	54.1	52.8
Health problems	50.9	44.7	38.7	62.5	57.4	66.7	50.5	52.8
Difficulty during delivery	46.3	45.5	51.6	60.4	31.1	53.3	44.4	48.8
Reduction of sexual feelings	25.2	27.2	29.0	12.5	24.6	13.3	27.3	20.8
Affects the health and welfare of women and girls	20.7	19.5	29.0	31.3	16.4	13.3	19.9	22.4
Difficult penetration during sex	4.0	2.4	3.2	12.5	1.6	6.7	4.6	3.2
No consequences	2.1	2.4	0.0	0.0	4.9	0.0	3.1	0.8
**Has seen a girl with complications after FGM/C**	40.9	33.2	41.5	60.3	39.7	55.0	33.8	51.1
**Considers FGM/C to be a mandatory religious practice (a)**	38.5	50.3	34.1	8.3	40.3	28.6	40.2	38.1

(a) For Ethnic group Chi-square = 35.88; P-value (0.000).

According to HCPs, FGM/C is mainly performed because people believe that the practice is mandatory by religion (53.8%), consider it to be deeply rooted in the Gambian culture (48.2%) and view it as an effective measure to reduce women’s sexual feelings (42.1%). Other given reasons include the fact that FGM/C is a rite of passage (34.4%), a good practice (23.3%), it helps to maintain virginity (13.5%), it reduces the rate of prostitution (11.1%), and does not violate human rights (0.6%).

Inter-ethnic analysis showed that the three most common answers for each ethnic group were the same found for the total group, showing a similar pattern of response. However, slight differences were found concerning the order of these three answers. Fula and Mandinka HCPs prioritised the fact that FGM/C is mandatory by Islam (58.7% and 54.9%), Serer and Wolof HCPs gave more importance to its cultural roots (80% and 55.3%), and Djola HCPs considered it to be important for attenuating sexual feelings (56.3%). Notably, significant differences were found among ethnic groups when HCPs were asked if they considered FGM/C to be mandatory by religion. While 50.3% of Mandinka HCPs answered affirmatively, only 8.3% of Wolof HCPs shared the same opinion (p = 0.000).

Inter-sex analysis showed that female and male HCPs also had similar opinions on the main three reasons given for FGM/C to be performed, although there were some nuances. More men than women considered that support towards FGM/C derives from the fact that it is mandatory by religion (56.3% vs. 48.8%) and attenuates sexual feelings (43.7% vs. 38.6%), whereas more women than men believed that people value the deep cultural roots of the tradition (55.1% vs. 43.7%). When asked if they consider FGM/C to be mandatory by religion, 40.2% men and 38.1% women responded affirmatively.

To evaluate HCPs’ knowledge on FGM/C-related complications, respondents were asked to identify five health consequences through an open-ended question. A considerable percentage of HCPs were able to recognise the negative impact of FGM/C on the health of girls and women. The transmission of infectious diseases was the most reported consequence (59.1%), which might be explained by the recent campaigns that international organisations working in The Gambia have launched for the prevention and treatment of HIV/AIDS. Bleeding (53.4%), difficulties during delivery (46.3%), and reduction of sexual feelings (25.2%) were also mentioned. Notably, 2.1% of HCPs, all of them of Mandinka and Fula origin, believed that the practice has no consequences. Inter-sex analysis showed that more women pointed out difficulties during delivery (48.8% vs. 44.4% men), while more men referred to the transmission of infectious diseases (57.6% vs. 42.4% women). Additionally, more men than women considered that FGM/C has no consequences (3.1% vs. 0.8%).

A considerable proportion of HCPs (40.9%) observed girls and women with health complications resulting from FGM/C. However, the rate of complications was substantially different among the ethnic groups. More than half of Wolof and Serer (60.3% and 55.0%, respectively) HCPs reported having seen a girl or woman with FGM/C complications, while this rate was 41.5% in Djola, 39.7% in Fula, and 33.2% in Mandinka HCPs. We found that a higher percentage of women than men (51.1% vs. 33.8%) answered affirmatively.

### Attitudes

Questions were designed to measure the attitude of HCPs towards the practice of FGM/C as follows: the feasibility of its elimination; different strategies to prevent FGM/C (including the role that can be played by Islamic leaders and by HCPs themselves); medicalisation; discrimination towards those who do not undergo FGM/C; and the involvement of men in the debate. The findings of attitude are shown in Table 3.

**Table 3:** Attitudes of Gambian health care professionals towards FGM/C

	**Total HCP (%)**	**HCP by ethnic group (%)**					**HCP by sex (%)**	
		**Mandinka**	**Djola**	**Wolof**	**Fula**	**Serer**	**Men**	**Women**
**Do you think that the practice of FGM/C should continue? (a)**								
Yes	42.5	57.3	39.0	20.7	42.9	23.8	45.7	37.4
No	57.5	42.7	61.0	79.3	57.1	76.2	54.3	62.6
**Do you think that girls that have not undergone FGM/C should be discriminated?**								
Yes	12.8	16.6	14.6	12.1	10.4	9.5	11.8	14.1
No	87.2	83.4	85.4	87.9	89.6	90.5	88.2	85.9
**Do you think that the practice of FGM/C can ever be eliminated in The Gambia?**								
Yes	45.2	39.2	31.7	64.3	38.2	64.7	43.4	48.6
No	54.8	60.8	68.3	35.7	61.8	35.3	56.6	51.4
**Do you think it is a good idea for men to be concerned on the debate on FGM/C?**								
Yes	78.3	69.5	85.4	89.8	83.3	90.5	80.5	75.0
No	21.7	30.5	14.6	10.2	16.7	9.5	19.5	25.0
**Do you think HCP workers have a role to play in eliminating FGM/C?(b)**								
Yes	73.0	59.8	70.0	89.7	80.5	90.0	70.2	77.4
No	27.0	40.2	30.0	10.3	19.5	10.0	29.8	22.6
**What do you think of ‘medicalizing’ FGM/C? (c)**								
It makes the practice safer	42.9	56.1	46.2	15.4	41.7	21.1	49.8	32.7
It is a way of encouraging FGM/C	16.5	17.7	15.4	11.5	20.8	10.5	17.7	14.8
It should be stopped at all levels	40.6	26.2	38.5	73.1	37.5	68.4	32.5	52.5

(a) For ethnic group Chi-square = 29.9; P-value = 0.000.

(b) For ethnic group Chi-square = 19.2; P-value = 0.002.

(c) For sex Chi-square = 16.49; P-value = 0.000.

A substantial percentage of HCPs (42.5%) believed that FGM/C should continue to be practiced, although this opinion was more commonly shared among traditionally practicing ethnic groups. The strongest support came from Mandinka (57.3%), Fula (42.9%) and Djola HCPs (39.0%), and the lowest support from Serer (23.8%) and Wolof HCPs (20.7%) (p = 0.000). A similar tendency was found in those believing that the practice can be eliminated, with the highest proportion in Serer (64.7%) and Wolof (64.3%), and the lowest in Mandinka (39.2%), Fula (38.2%), and Djola HCPs (31.7%). Inter-sex differences also existed. Men were more supportive towards FGM/C than women (45.7% vs. 37.4%), and less confident on the feasibility of it being abandoned (43.4% vs. 48.6%).

Regarding strategies to prevent the practice, Mandinka, Djola, and Fula HCPs were less eager for the idea of religious leaders preaching against FGM/C, with this strategy finding the strongest support among Wolof (71.2%) and Serer HCPs (76.2%). When asked if HCPs have a role to play in eliminating FGM/C, 73.0% of the respondents answered affirmatively. However, while the majority of Wolof and Serer HCPs (89.7% and 90%, respectively) welcomed this idea, Mandinka HCPs were not that supportive (59.8%) (p = 0.002). When these results were examined for a difference between sexes, women appeared to be more favourable than men to this suggestion (77.4% vs. 70.2%).

Table 3 shows that 42.9% of all HCPs considered medicalisation as a safer practice, compared with how the cutting is traditionally performed by a circumciser, and this belief was more prevalent among Mandinka (56.1%), Djola (46.2%), and Fula HCPs (41.7%) than the other ethnic groups. However, 16.5% of the respondents viewed medicalisation as a way of encouraging FGM/C and 40.6% defended that it should be stopped at all levels. Wolof and Serer HCPs showed the highest support to stop medicalisation (73.1% and 68.4%, respectively), while only one fourth of Mandinka HCPs (26.2%) agreed with this idea. Women, more than men, stand against medicalisation (52.5% vs. 32.5%, p = 0.000).

Discriminatory attitudes towards those who do not undergo FGM/C were found in 12.8% of HCPs, and this attitude was the most common among Mandinka and Djola HCPs (16.6% and 14.6%, respectively). When these results were examined for a difference between the sexes, women had a more discriminatory attitude than men (14.1% vs. 11.8%). However, when inter-sex analysis was conducted within the ethnic groups, an exception was found for Mandinka and Djola HCPs, where men’s support for discrimination was slightly higher than that for women.

A high percentage of HCPs (78.3%) considered that men should be concerned about the debate on FGM/C. Serer and Wolof HCPs expressed the strongest support for men’s involvement (90.5% and 89.8%, respectively), followed by Djola (85.4%), Fula (83.3%), and Mandinka (69.5%). This opinion was also more popular among men than in women (80% vs. 75%).

### Practices

The practices concerning FGM/C were assessed by investigating if FGM/C was performed in the HCPs’ family/household, if they intended to subject their own daughters to FGM/C, and if they had ever performed FGM/C during their medical practice. The results for practices are shown in Table 4.

**Table 4:** Practices of FGM/C among Gambian health care professionals

		**Total HCP (%)**	**HCP by ethnic group (%)**					**HCP by sex (%)**	
			**Mandinka**	**Djola**	**Wolof**	**Fula**	**Serer**	**Men**	**Women**
**Is FGM/C practiced in your family/household?**	Yes	68.6	85.7	85.0	19.0	63.3	42.9	70.8	58.5
	No	31.4	14.3	15.0	81.0	36.7	57.1	29.2	41.5
**If you have a daughter in the future, do you intend to circumcise her? (a)**	Yes	47.2	64.3	47.5	8.6	43.6	23.8	48.8	40.8
	No	52.8	35.7	52.5	91.4	56.4	76.2	51.2	59.2
**As a health care provider, have you ever carried out FGM/C on a girl?**	Yes	7.6	8.0	9.8	5.3	6.5	5.0	6.9	7.4
	No	92.4	92.0	90.2	94.7	93.5	95.0	93.1	92.6

(a) For ethnic group Pearson Chi-squared = 62.1; P-value = 0.000.

A total of 68.6% of HCPs reported that FGM/C is practiced in their family/household. Inter-ethnic analysis showed that the rate of prevalence of FGM/C in HCPs’ families/households was comparable with the last available figures, published by UNICEF in The Gambia [18], strengthening the credibility of our study’s results. FGM/C was practiced among the families/households of 85.7% Mandinka, 85.0% Djola, 63.3% Fula, 42.9% Serer and 19.0% Wolof HCPs. Even though the intention of HCPs to carry on with the practice of FGM/C, by subjecting their own daughters to it, was considerably less than the rate of FGM/C in their family/household, this rate was still high, particularly among Mandinka (64.3%), Djola (47.5%), and Fula HCPs (43.6%) (p = 0.000).

When these practices were examined in both sexes, more men than women assumed that FGM/C is practiced in their families/households (70.8% vs. 58.5%), and admitted their intention to have it performed in their daughters (48.8% vs. 40.8%). This intra-sex tendency was maintained throughout all ethnic groups, except for Fula and Serer, among whom more women expressed this desire. An inverse relationship was found between HCPs’ reported rates of intention to subject their daughters to FGM/C and rates of exposure to FGM/C health consequences.

Finally, we also found that medicalisation was a reality in The Gambia, with 7.6% of HCPs admitting to having performed FGM/C in girls. This was more frequent among Djola (9.8%) and Mandinka HCPs (8.0%) than among other ethnic groups. More women than men carried out FGM/C during their medical practice (7.4% vs. 6.9%).

## Discussion

Our study showed that a considerable proportion of Gambian HCPs working in rural areas embraced the continuation of FGM/C. They also intended to carry on with this practice by subjecting their own daughters to FGM/C, with some of them reporting to have already performed it during their medical practice. However, their KAP are, to a large extent, shaped by sex and ethnic identity.

### Intra-sex analysis

While the majority of the intra-sex differences in this study were not significant, they still showed the intricate web of meaning that surrounds the practice of FGM/C. FGM/C had lower support among women than among men. Indeed, female HCPs showed less approval for continuation of FGM/C, more confidence on the feasibility of its abandonment (48.6% vs. 43.4%), higher endorsement of the proposed strategies to prevent it, and lower intention to have it performed on their own daughters than male HCPs. While women appear to be willing to overcome their subordinate position in a patriarchal society that suppresses their right to make decisions on their own body, they are undermined by the coercive impact of the socio-cultural norms. We found that women exhibited a more discriminatory attitude towards girls who had not undergone FGM/C and were more aware than men of the powerful force that lies beneath its tradition, when considering its deep cultural roots as the main reason for its perpetuation.

This higher consciousness in women can be explained by the fact that women have contributed to embed the practice of FGM/C into a deep social and female meaning. Within patriarchal societies, women have coped with repression by adding value to the elements that configure it, eventually building their identity in them. In this sense, FGM/C grants women a special value, by attesting their femininity and making them worth inclusion in their social network. This value is lacking in those who do not undergo FGM/C, and justifies their discrimination. This interpretation has been confirmed in a previous study carried out in The Gambia, among the general population [35]. This previous study showed that the current FGM/C practices are more accurately explained by the “peer convention” hypothesis than by the “marriage convention” hypothesis, because of the fact that they give women a capital social value that empowers them in the society, building them a social identity.

Because women’s gender identity depends on this special value, women ensure its transmission in a ceremony that encloses the practice of FGM/C with secrets, and FGM/C becomes part of the “women’s world”. This might explain the highest resistance of female HCPs to the inclusion of men in the debate on FGM/C. This may also be connected with the differences found regarding the awareness of the health consequences of FGM/C. In our survey, more female than male HCPs were aware of the existence of these consequences and have actually observed them in patients. Male and female HCPs receive the same training at health schools, but by the time that this study was conducted, FGM/C was not included in their academic curriculum. Therefore, a plausible explanation for the increased awareness revealed by women is that they, by knowing how FGM/C is performed, are also able to realise how it changes female genitalia. Therefore, women can more easily understand than men that this change has a connection with the health problems of their patients. When female HCPs become aware of the health complications that derive from the practice, the struggle begins on whether to let the secret surrounding FGM/C hide these health consequences, or, instead, choose to stand against it, facing the risk of being set apart from their community.

With regard to men, FGM/C appears to be viewed from a moral perspective. They prioritise the fact that this practice is mandatory by religion and attenuates women’s sexual feelings, contributing to family honour. However, although a new line of research on how men perceive and relate to FGM/C has already been initiated, men’s KAP towards this “female” practice have scarcely been addressed. Further investigation in this area is required to understand men’s position on this issue and develop strategies to promote their involvement. Our finding that 80.0% of male HCPs considered that men should participate in the debate surrounding FGM/C, suggests their willingness to do so.

### Inter-ethnic analysis

Our finding of significant differences among ethnic groups show how strongly ethnic identity shapes the KAP of HCPs. In line with the most recent studies conducted in The Gambia [13,33,36], our findings showed that FGM/C is not homogeneously practiced. The prevalence of FGM/C is higher among Mandinka, Djola and Fula, which are traditionally practicing groups. They are also groups who more strongly embrace the continuation of FGM/C, express more scepticism about its eventual elimination, and are less supportive of prevention strategies than the other groups.

The low, but still significant rate of prevalence of FGM/C found among Wolof and Serer, who traditionally do not perform FGM/C, indicate a phenomenon that is particularly common in rural areas, such as the regions examined in this study, the assimilation of other’s ethnic identity. Despite the fact that different ethnic groups might share the same territory, the identity of the major ethnicity prevails. For the smaller groups, incorporation of this identity is vital because the sense of community is one of the core values upon which African social life is based [37]. Everyone wants, and needs, to belong. For this reason, FGM/C has been adopted by traditionally non-practicing groups as part of this larger process of integration and assimilation that started, frequently through intermarriage, once they settled in communities where FGM/C is a tradition. Therefore, it is understandable that Wolof and Serer HCPs were found to be the most prominent groups considering the deep cultural roots of tradition as the major reason for the perpetuation of FGM/C.

With regard to Fula and Mandinka HCPs, the mandatory requirement according to religion primarily encourages and legitimises the continuation of FGM/C. However, this connection between Islam and FGM/C is not consensual among HCPs, despite the fact that they all come from ethnic groups with Muslim affiliations. When HCPs were asked directly if they considered FGM/C to be mandatory by religion, half of Mandinka HCPs answered affirmatively, while very few Wolof HCPs shared the same opinion. Mandinka HCPs are also less supportive towards having religious leaders preaching against FGM/C, which is in contrast to Wolof and Serer HCPs who are more supportive. These findings suggest that tradition and ethnic identity have the power to shape the way religion is understood and interpreted.

Ethnic identity also confines the intention expressed by HCPs to have FGM/C performed on their own daughters. This intention was found to be much higher among Mandinka than among Wolof HCPs. Interestingly, not all HCPs who were part of families who practice FGM/C intended to perpetuate it in their descendants. A considerable decrease was observed between the rates of reported practice of FGM/C in the family/household of HCPs and the intention of future practice on their daughters. This rupture with family tradition might be a consequence of the global social change in local traditions, frequently brought up by community members living abroad. The effect of migration on KAP of local populations is a promising field of study that should be explored for better understanding of the factors underlying social change. However, a complementary (and perhaps stronger) explanation can be found in HCPs’ level of education. This interpretation is supported by previous studies that have shown an association of FGM/C practices with low education levels [37,38]. In The Gambia, data collected in 2006 by the Gambia Bureau of Statistics from a general population sample of girls and women (15–49 years) [39] showed a much higher rate of intention to practice FGM/C (72.9%) than that found in this study of female HCPs (40.8%), which appears to support this interpretation.

Education is indeed a recognized powerful tool for promoting change. However, the fact that almost all HCPs are aware of FGM/C health complications and that a substantial amount (42.5%) still support this practice, suggest that professional training should be carefully designed. Several examples found in this study indicate that ethnic identity appears to bias knowledge and perceptions, and this should be taken into consideration in training programmes. HCPs who denied that the practice of FGM/C has health consequences were of Mandinka and Fula origin. However, more than half of Serer and Wolof HCPs had seen a girl or woman with FGM/C complications, and only 33.2% of Mandinka, 41.5% of Djola, and 39.7% of Fula HCPs reported observing complications. The acceptance of this reality is clearly modelled by HCPs’ ethnic background. In line with our discussion above, ethnic identity appears to prevail over professional identity.

The finding that only 40.9% of all HCPs had seen a girl with FGM/C-related complications raises concern, taking into consideration that an estimated 76.3% of Gambian girls and women have been subjected to this practice, and that in some regions, FGM/C prevalence rates are almost universal (99% in Basse, Upper River Region). A lot more HCPs might have been exposed to FGM/C health consequences without being able to connect the complications with this practice. This is indicative of the gaps in the professional education of HCPs, and reinforces the need of building their capacity in the identification, management and prevention of FGM/C through quality training.

### The risk of medicalisation and the role of HCPs

This study showed that medicalisation is already a reality in The Gambia, as it is in other African countries, with 7.6% of HCPs confirming to have performed FGM/C on girls. This was reported by male and female HCPs, showing that, in a medical setting, FGM/C is no longer reserved only to women. Moreover, there was support for medicalisation for a considerable proportion of HCPs because 42.9% of them believed it was an alternative to make the practice safer.

Medicalisation has been publicly condemned by WHO because it creates a sense of legitimacy, gives the erroneous impression that the practice is harmless, and represents a break in medical professionalism and ethical responsibility [36]. Notwithstanding, significant forces of the Gambian social context have been encouraging this tendency. Throughout the past years, The Gambia has had a growing influence of conservative Islamic leaders over authorities and parliamentarians [12], and some of these leaders promote medicalisation. This was confirmed in a recent encounter for policy dialogue between religious leaders and international organisations working towards the prevention of FGM/C, organised by the Gambian Women’s Bureau, and attended by one of the authors, in September 2012.

In these circumstances, it is not surprising to find that the highest support of medicalisation came from those HCPs who relate FGM/C to religion. This was particularly evident for the Mandinka group, where more than half of them perceived medicalisation as a safer practice and only one quarter defended that it should be stopped at all levels. Mandinka respondents were also less eager regarding the idea of having HCPs playing a role in eliminating FGM/C. The rate of support of Mandinka HCPs (59.8%) was markedly different to that found among the non-traditionally practicing groups (Wolof, 89.7%; Serer, 90%).

## Conclusion

Our findings show a concerning rate of support towards FGM/C among Gambian HCPs, as well as a tendency for medicalisation. Our study also shows the ignorance of HCPs regarding FGM/C health consequences, because with an overall prevalence of 76.3% for FGM/C, which increases almost up to universal rates in some regions, only 40.9% have observed FGM/C-related complications. In these circumstances, these results indicate a critical and urgent need to develop effective strategies to build the capacity of HCPs to prevent the practice, ensure proper management of its consequences, and avoid medicalisation.

However, capacity-building strategies should be carefully designed because education per se is not a guarantee for FGM/C abandonment. To be effective, training programmes must be culturally and gender sensitive, being modelled to fit the specific characteristics of the trainees in terms of sex and ethnicity. Likewise, the finding that there is an inverse relationship between the rate of exposure to FGM/C health consequences and the rate of intention to subject daughters to this practice, suggests that training programmes, which include a component of exposure, can become a powerful strategy for prevention.

Gender inequity remains a reality, and it is fundamental to acknowledge the strategies found by women to cope with it. The complexity of the FGM/C issue is evident from the contradictory positions assumed by the women in this study, and these must be recognised and addressed. However, from a gender perspective, social change can be effective only by taking into consideration of the role played by both men and women. Therefore, a deeper understanding of men’s KAP is urgently required.

Moreover, it is important to acknowledge how ethnic identity tends to prevail over professional identity. HCPs belonging to traditionally practicing ethnic groups are more willing to perpetuate FGM/C, either by having it performed on their descendants, or by performing it themselves throughout their medical practice. Traditionally practicing ethnic groups are also the most reluctant to admit a role for HCPs in elimination of FGM/C. Nevertheless, HCPs have the potential to become important agents for the prevention of FGM/C. HCPs are integrated and legitimated in the community. HCPs are also on the first line of response for FGM/C-related complications. The involvement of HCPs is particularly urgent in rural areas, where the prevalence of FGM/C is higher than in urban areas, and several constraints hinder the access to quality health services, which is likely to increase the magnitude of FGM/C health consequences in the lives of girls and women.

Because this is the first study of this type conducted in The Gambia, the results have the potential to be used as a baseline for assessing eventual changes in the KAP of HCPs, after implementation of a training programme.

## Endnotes

^a^Infant mortality is estimated at 81 per 1000, and under-5 mortality is at 109 per 1000 [13].

^b^Gambian women, who have an average birth rate of 5 children, face the highest risk of death in pregnancy, delivery, or postpartum, of the whole region [12].

## Competing interests

The authors declare that they have no competing interests.

## Authors’ contributions

AK performed the previous studies on FGM/C, proposed and designed the present KAP study, coordinated the data collection, participated in data handling and analysis, and reviewed the manuscript. SH participated in the design of the study, collaborated in the training of Community-based Medical Programme students for social research techniques, and supervised the fieldwork. MB conducted data analysis and statistical calculations, and drafted the article. IB participated in data analysis, manuscript review, and the publication process. All authors read and approved the final manuscript.

## Pre-publication history

The pre-publication history for this paper can be accessed here:

http://www.biomedcentral.com/1471-2458/13/851/prepub
